# Poppers Use and High Methaemoglobinaemia: ‘Dangerous Liaisons’

**DOI:** 10.3390/ph14101061

**Published:** 2021-10-19

**Authors:** Malcolm Barrangou-Poueys-Darlas, Marie Gerardin, Sylvie Deheul, Marion Istvan, Marylène Guerlais, FAN, Pascale Jolliet, Thomas Dejoie, Caroline Victorri-Vigneau

**Affiliations:** 1 Centre for Evaluation and Information on Pharmacodependence, Clinical Pharmacology Department, Nantes University Hospital, 9 Quai Monsousu, 44000 Nantes, France; malcolm.barrangoupoueys@chu-nantes.fr (M.B.-P.-D.); marie.gerardin@chu-nantes.fr (M.G.); marion.istvan@chu-nantes.fr (M.I.); marylene.guerlais@chu-nantes.fr (M.G.); pascale.jolliet@univ-nantes.fr (P.J.); 2 Centre for Evaluation and Information on Pharmacodependence, Clinical Pharmacology Department, Lille University Hospital, 1 Place de Verdun, 59037 Lille CEDEX, France; sylvie.deheul@chru-lille.fr; 3 INSERM UMR 1246 SPHERE (Methods in Patients-Centered Outcomes and HEalth Research), Nantes and Tours Universities, 44000 Nantes, France; 4 Department of Biochemistry, University Hospital of Nantes, 44000 Nantes, France; thomas.dejoie@chu-nantes.fr

**Keywords:** poppers, abuse, methaemoglobinaemia, toxicity

## Abstract

Poppers are legal and largely used in France despite severe side effects, such as methaemoglobinaemia (MetHbia). Our work aimed to assess the prevalence of poppers consumers among patients with a MetHbia higher than or equal to 5% in French university hospitals and its evolution before and after the legalization of poppers in France. We conducted a national multicentre observational retrospective study. All patients for whom at least one MetHbia measurement was performed from 2012 to 2017 in university hospitals where the French addictovigilance network (FAN) is implanted were included. For each MetHbia measurement exceeding or equal to 5%, a return to the clinical file was made by the FAN to assess poppers consumption. We calculated the prevalence of MetHbia exceeding or equal to 5% and 25% and the prevalence of poppers consumption before and after the legalization. A total of 239 (0.14%) patients had a MetHbia level exceeding or equal to 5% with 25 (10.46%) cases of poppers consumption. Poppers consumption represented 68.4% (13 out of 19) of cases with MetHbia greater than or equal to 25%. Poppers consumption among patients with MetHbia exceeding or equal to 5% increased after the legalization from 4.76% to 11.67% (prevalence ratio PR = 2.45, 95% CI = [0.98–8.37], *p*-value = 0.190). The proportion of patients with a MetHbia level of 25% or more increased after the legalization from 4.76% to 8.63% (PR = 1.81, 95% CI = [0.68–6.82], *p*-value = 0.374). The use of poppers is very frequently reported by patients with MetHbia greater than or equal to 25%.

## 1. Introduction

Poppers or alkyl nitrites are volatile substances sold in small glass bottles and are largely used as recreational substances in France. In 2017, poppers was the second most experimented illicit substance among 18–64 year olds, after cannabis, with a prevalence of 8.7% of the people concerned [1].

Organic nitrites, such as alkyl nitrites, are partially metabolized in the liver to nitrogen monoxide, which is a powerful cerebral and peripheral vasodilator. They were first used as a treatment for angina pectoris due to their vasodilatory effects [2] before being replaced by other nitro compounds, including trinitrine. Currently, they no longer exist as medicines. However, the increasing use of this substance in festive circles for a diverse range of uses makes it a frequently reported and commonplace product. The French Monitoring Centre for Drugs and Drug Addiction (*Observatoire français des drogues et toxicomanies,* OFDT) reported massive consumption among adolescents and young adults looking for euphoric effects [3]. Electro club regulars use them to potentiate the effects of other stimulants, mainly cocaine and MDMA, while people from the LGBTQ community usually consume this product to stimulate libido and facilitate sexual practices [4]. Regular use is concerning, in particular, subjects of the male homosexual population going to festive or sexual meeting places. In this context, some users feel almost dependent on the product to perform sexual acts [1]. For example, since 2014, in Australia, there has been a significant increase in the use of poppers among gay, bisexual, and other men who have sex with men [5].

In addition to the effects induced by its vasodilating properties, especially headache, flush, and cardiovascular effects, the consumption of poppers can induce nausea, vomiting, mental disorders, and many potentially serious side effects, such as maculopathies [6] and blood disorders, especially cyanosis and methaemoglobinaemia (MetHbia). Indeed, metabolization of organic nitrites by the liver is known to cause oxidation of hemoglobin to MetHb, increasing its concentration in blood above physiological levels [7]. MetHbia should not exceed 1 or 2% in the physiological state [8]. Above a particular methemoglobinemia threshold, this can lead to cyanosis and hypoxia [9]. In 2011, a review of MetHbia cases associated with poppers consumption reported MetHbia ranging from 17.8% to 94% [2]. Since this review, around the world we have observed an increasing number of case reports in the literature alarming us about the side effects of poppers consumption [10,11,12,13,14,15].

The French National Agency for the Safety of Medicines and Health Products (*Agence nationale de sécurité du médicament et des produits de santé*, ANSM) is in charge of poppers monitoring through the French Addictovigilance Network (FAN) [15]. The FAN is a network of 13 Drug Dependence Evaluation and Information Centres (*Centre d’évaluation et d’Information sur la Pharmacodépendance—Addictovigilance*, CEIP-A) throughout France. The three main CEIP-A missions are to collect data and assess the dependence potential of identified psychoactive drugs, provide information on the risk of abuse or dependence on psychoactive substances, and perform research [15].

Over the past two decades, the regulation of popper sales has changed several times in France. It was prohibited by three different decrees/orders (decree n° 90–274, 26 March 1990; decree n° 2007–1636, 20 November 2007; and order of 29 June 2011) that were successively cancelled by the Council of State and the Ministry of Economy and Finance [16]. The ANSM first conducted an assessment of the risks of poppers abuse in 1999, which led to the 2007 decree prohibiting poppers sales and distribution. The Council of State on 15 May 2009, cancelled this decree. On 21 April 2011, the ANSM once more highlighted the risks of poppers abuse and was in favor of banning poppers sales and distribution altogether. The Ministry of Health prohibited the sale and distribution to the public by a bylaw (29 June 2011), but this decree was cancelled by a decision of the Council of State (Decision of the Council of State n° 352484 and 352485; 3 June 2013). Since then, poppers have been sold freely.

The ANSM has since regularly renewed the assessment of the risks of poppers abuse. These reports showed an increase in spontaneous notifications for poppers, and all FAN tools have detected signals of poppers abuse [17,18]. In this context, the use of the MetHbia measurements routinely performed in the context of inpatient care was found to be relevant obtain comprehensive surveillance of one of the most serious side effects. The MetHbia measurement represents an indirect tool for screening serious problematic poppers consumption in symptomatic patients who need medical attention and was tested in a previous local study with a defined threshold greater than or equal to 5% [19].

The main objective of our national study was to assess the prevalence of poppers consumers among patients with a MetHbia higher than or equal to 5% in French university hospitals. We also described the evolution of the prevalence of poppers consumers before and after the legalization of poppers in France and the clinical characteristics of these consumers.

## 2. Results

### 2.1. Stages of the Study

**Figure 1:**
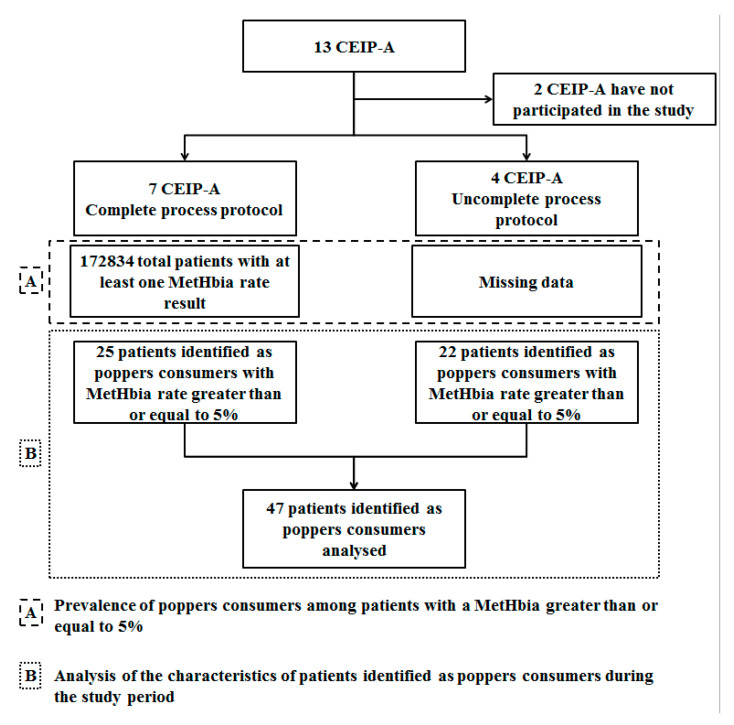
Flow chart of CEIP-A participating in the study and identified popper consumption cases. CEIP-A: Drug Dependence Evaluation and Information Centres (Centre d’évaluation et d’Information sur la Pharmacodépendance—Addictovigilance). MetHbia: Methaemoglobinaemia.

Eleven CEIP-A participated in the study. Nevertheless, 7 of 13 had a complete process protocol as shown in Figure 1. Data from these CEIP-A were used for part A of the study. Cases of poppers consumption among patients with an elevated MetHbia level used for part B of the study were transmitted by the 11 CEIP-A.

#### 2.1.1. Part (A): Prevalence and Evolution of the Number of Cases of Poppers Consumers among Patients with a MetHbia Greater than or Equal to 5% Assessment

##### Primary Outcome

Over the entire study period, a total of 172,834 patients were identified through the results of 758,348 MetHbia tests according to the study protocol. Among all patients in the study, 239 (0.14%) patients had a MetHbia rate greater than or equal to 5%, with 25 (10.46%) cases associated with poppers consumption reported in the medical record.

##### Evolution of the Prevalence before and after the Legalization of Poppers in France

Table 1 shows the number of cases of poppers consumption among patients with a MetHbia result greater than or equal to 5% identified in the total study period and by period. It suggests an increase in poppers consumption among patients with MetHbia levels greater than or equal to 5% after the legalization of poppers in May 2013 from 4.76% to 11.67% (PR = 2.45, 95% CI = [0.98–8.37], *p*-value = 0.190). While the proportion of patients with a MetHbia rate greater or equal to 5% was stable over the study period, the proportion of patients with a MetHbia rate of 25% or more seemed to have an increasing trend after the legalization of this product from 4.76% to 8.63% (PR = 1.81, 95% CI = [0.68–6.82], *p*-value = 0.374).

In addition, we note that just after the change in regulations, the proportion of poppers users among patients with a MetHbia greater than or equal to 5% tripled and the proportion of MetHbia greater than or equal to 25% doubled.

##### Proportion of Poppers Consumers (Reported in Medical Records) According to the MetHbia Level

The number of poppers cases was not evenly distributed across MetHbia levels. Poppers consumption represented 5.5% (12 out of 220) of cases with MetHbia greater than or equal to 5% and 68.4% (13 out of 19) of cases with MetHbia greater than or equal to 25%.

#### 2.1.2. Part (B): Description of the Characteristics of Cases of Patients Identified as Consuming Poppers during the Study Period

The average age of the subjects was 35 (10.3). Among poppers consumers, the range of recorded MetHbia levels was wide, from 5.4% to 87.8% (Table 2). In 57% (27 out of 47) of the cases of poppers consumption, the MetHbia rate was greater than or equal to 25%. Medical intervention required methylene blue in 68% and oxygen therapy in 57% of cases. We found that eight patients had severe side effects, and a majority of them had a MetHbia rate lower than 25%. Cyanosis, consciousness disorder, and malaise, as well as the use of oxygen therapy and methylene blue, seemed more frequent in the group with MetHbia levels greater than or equal to 25% compared to the group with levels lower than 25%. The amounts of poppers consumed were rarely reported (21 out of 47 identified poppers consumers reported this information), although this may be clinically relevant information. When this was reported, it ranged from four breaths in a single dose to multiple episodes of intake over several hours.

## 3. Discussion

The main objective of our study was to assess the prevalence of consumers of poppers among individuals with MetHbia rates greater than or equal to 5% in French university hospitals. To our knowledge, this is the first study on poppers consumption and the abnormally high rate of elevated MetHbia levels at the national level in France. A high prevalence of MetHbia greater than or equal to 25% was associated with the use of poppers: poppers consumption represented 5.5% (12 out of 220) of cases with MetHbia greater than or equal to 5% and 68.4% (13 out of 19) of cases with MetHbia greater than or equal to 25%.

Even if the increase in consumption, particularly since the legalization of poppers in France in June 2013 is, in our study, only a trend, the results showed more than a doubling from before to after the legalization. This increase was also reflected in the number of patients with MetHbia levels greater than or equal to 25%, which has also doubled. The number of consumers of poppers identified in our study was not sufficient to obtain significant results despite the collection of data at the national level. However, the large variation observed before and after popper legalization in the number of abnormally high MetHbia levels and the over-representation of poppers consumers in this category suggest that if this study was replicated over a longer period of time, we would obtain significant results.

Moreover, the consumption of poppers has appeared to have become more widespread and commonplace in France [17]. Increasing availability via legalization can increase the use of a product [20]; this kind of effect has already been observed with other substances, such as cannabis in Canada, after its legalization. Indeed, the national cannabis survey found that 18% of Canadians aged 15 years and older reported using cannabis in the first quarter of 2019, and this proportion was higher than the 14% reported one year earlier, before the legalization [21].

The frequency of oral consumption of poppers was surprisingly high, which was relatively unknown to the FAN. Indeed the most frequently reported mode of administration is the inhaled route [1]. The existing literature mentioned alarming cases of intoxication in the context of unintentional or accidental ingestion, as has already been described in France [22], but, to our knowledge, little was reported in the literature. The fact that we found several cases in our study leads us to wonder about a possible greater oral toxicity. The monitoring of cases will allow us to confirm this hypothesis.

The dermal toxicity of poppers is known in the context of inhaled consumption [23] but less in the context of ingestion. Oral consumption of poppers seems to cause more serious overall patterns with higher levels of MetHbia. In our results, the median and maximum measured MetHbia levels were probably underestimated, with two out of 10 cases of oral consumption having MetHbia levels higher than the calculation capacity of the device used.

In cases involving the use of substances associated with poppers, the risk of toxic effects theoretically increases. We cannot show this from our data, but as an example, concomitant consumption of sildenafil and nitrates in any form drastically increases the pharmacological risk of hypotension. Indeed, the administration of nitrate medications with a phosphodiesterase type 5 inhibitor represents an absolute contraindication in the concerned drug approvals.

Similarly, in patients with no coronary history who had a first angina episode after sexual activity, the possible use of a phosphodiesterase type 5 inhibitor (most often in the hour before sexual activity) should be investigated, by questioning, and if this was the case, abstain from any nitro-treatment. Moreover, co-administration of poppers with other psychoactive substances, such as cocaine, can induce or enhance the side effects inherent in these products in addition to the cardiovascular risk specific to poppers. Cocaine itself and its metabolites are not recognized to cause MetHbia [2], but some adulterants added to cocaine have been associated with methemoglobinemia.

Inter-individual differences in psychoactive drug use are an inevitable source of bias. For example, with tobacco, due to the lack of a low-cost estimation of the exact quantity of inhalations per cigarette, we estimate tobacco consumption in pack-years. From one smoker to another, many parameters are taken into account, such as the way a cigarette is smoked, the parameters of the inhalation itself, or the type of tobacco consumed. A similar consideration applies to the consumption of poppers. This product is usually inhaled by applying a nostril close to the opening of the vial; however, depending on the particular method, two different consumers with different consumption patterns will not have consumed the same amount of product for an equal number of inhalations. From one consumer to another, the effects felt and the risks incurred can vary greatly.

A question persists about the high variability in MetHbia levels found in our population. Although inter-individual variability probably had an impact, as well as the consumption pattern, the nature of the product consumed itself remains a key element. Products from the same pharmacological class or close structure can vary greatly in terms of potency and adverse effects once administered and metabolized by the body. This inter-product variability remains insufficiently described and studied at present.

MetHbia-induced tissue hypoxia can lead to severe or even fatal complications. The clinical effects of methemoglobinemia can usually be predicted from the methemoglobin concentration [2]. However, patients with comorbidities that decrease oxygen transport or delivery (respiratory and/or cardiovascular disease or anemia) can develop significant symptoms at lower MetHbia concentrations, which could explain our results regarding medical intervention in consumers presenting relatively low MetHbia rates. Nevertheless, to confirm this hypothesis, an analysis of data on the comorbidity or underlying illness of the patients with a severe clinical profile and low MetHbia rates would be helpful. We observed that most patients (68%) with a MetHbia level greater than or equal to 25% were reported as poppers consumers and that the combined increase in poppers consumption cases and MetHbia levels greater than or equal to 25% was related.

The percentage of poppers consumers among patients was likely underestimated in this study. The search for poppers consumption was performed retrospectively in medical records. It is likely that many cases of poppers use were not mentioned in medical records because the use of psychoactive substances is generally not sought after in the history taking. However, despite this limitation, the metrological characteristics of this method as a screening and monitoring tool are relatively good. Using the information from our study, healthcare professionals are encouraged to keep in mind the diagnostic hypothesis of poppers consumption in situations with elevated MetHbia levels.

The principal limitation of this type of collection lies in the application of a common methodology to structures of very different sizes and functioning. Complete data were collected in 7 of the 13 CEIP-A. The total number of patients with assessed MetHbia levels collected in university hospitals varied enormously from one hospital to another, with some performing this measurement systematically in blood gases even in non-symptomatic patients. These differences reflect very different practices in MetHbia testing and recording of results.

Moreover, we only have data for one year of “illegal poppers”. Indeed, in designing the study, we wanted to have baseline data when poppers were illegal, but more importantly, we wanted to be able to assess the evolution after the change. As we could not conduct the study for too long a period given the work involved, we chose a total of five years.

As poppers consumption was identified from medical records, we can conclude that the legalization could have influenced the reporting. Indeed, patients may be more likely to admit to the consumption of a legal substance than the consumption of an illegal one, which could contribute to the increasing number of consumers. Additionally, even if patients report consumption, they may be under-reporting the amount consumed.

Finally, although this is a national study, the absolute number of poppers users identified among the patients is small and fluctuates. A longer study including more university hospitals would be useful to confirm the trends.

## 4. Methods

### 4.1. Study Oversight

This study was divided into the following parts:

Part (A) was a national observational retrospective study conducted by FAN in French university hospitals from 1 January 2012 to 31 December 2017, to assess the prevalence of poppers consumers among patients with a MetHbia higher than or equal to 5%. We described this prevalence at different time periods: the period before the change in the regulation of poppers sales (January 2012 to May 2013) and the period after this change.

Part (B) was an analysis of the characteristics of identified patients consuming poppers during the study period.

### 4.2. Patients

The study population included patients having at least one MetHbia measurement performed from 1 January 2012, to 31 December 2017, in the university hospitals where the CEIP-A were located.

We identified poppers consumers (consumption associated with MetHbia registered in the medical record) among patients with at least one MetHbia higher than or equal to 5%.

### 4.3. Collected Data

#### 4.3.1. Number of Patients with MetHbia Measurement

The total number of patients who had at least one MetHbia measurement, the number of patients who had a MetHbia result greater than or equal to 5%, and the number of patients who had a MetHbia result greater than or equal to 25% were collected. The MetHbia measurements were obtained from arterial or venous samples performed as part of the usual management of patients and analyzed according to standardized conditions in each laboratory of the university hospitals with a CEIP-A participating in this study. In the context of inpatient care, several MetHbia measurements were possibly performed for the same patient.

#### 4.3.2. Number of Poppers Consumers among Patients with a MetHbia Greater than or Equal to 5%

A return to the clinical records was performed to look for additional information: poppers consumption associated with the hospitalization in which patients had a MetHbia greater than or equal to 5%, symptoms, clinical complications, medical support, and clinical evolution.

### 4.4. Ethics

According to French legislation, this study did not require ethics committee approval.

### 4.5. Outcomes

In the population with a MetHbia greater than or equal to 5%, our primary outcome was the number of patients consuming poppers identified in the medical records in the overall study period.

Secondary outcomes were (i) the evolution of the prevalence of patients consuming poppers before and after the legalization of poppers in France, (ii) the proportion of poppers consumers according to the MetHbia level, and (iii) the sociodemographic characteristics and the clinical profile of patients identified with poppers consumption according to the MetHbia level.

### 4.6. Statistical Analysis

Descriptive results were expressed using the mean and standard deviation or median and interquartile range for continuous variables and count and percentage for categorical variables.

The prevalence of patients with a MetHbia level greater or equal to 25% and the prevalence of poppers consumers according to the report in medical records among patients with a MetHbia level greater or equal to 5% were calculated before and after the regulatory change using a quasi-Poisson regression model. The results are presented as a prevalence ratio (PR) and a 95% confidence interval (95% CI).

## 5. Conclusions

Poppers toxicity and potential severe side effects cannot be ignored. We focused our study on the relationship between poppers consumption and elevated MetHbia levels, but we should not forget other serious complications linked to the consumption of this product, such as maculopathies [6,24] or complications induced by the powerful vasodilating effect of poppers. Poppers consumption represents a non-negligible danger on the scale of a population but also at the individual level. A total of 25 patients out of 239 does not seem high from a population point of view. Nevertheless, it should be borne in mind that our data do not reflect the entire consumption of poppers, nor all the undesirable effects, but target a very serious effect.

It is necessary to inform as widely as possible about the hazards of using poppers. The fact that their sale is no longer regulated can give the impression of a trivial non-hazardous substance, when in fact it is not.

## Figures and Tables

**Table 1:** The number of poppers consumption cases identified in the total study period and by period among patients with a MetHbia level ≥ 5% included in the study protocol.

	Total	Before Regulation Change	After Regulation Change				
n (%)	January 2012 to December 2017	From January 2012 to May 2013	From June 2013 to December 2014	From January 2015 to December 2015	From January 2016 to December 2016	From January 2017 to December 2017	From June 2013 to December 2017
Total number of patients	172,834 (100%)	30,480 (17.63)	39,817 (23.04)	30,117 (17.42)	33,164 (19.19)	39,196 (22.68)	142,354 (82.36)
Total number of patients with MetHbia ≥ 5%	239 (0.14)	42 (0.14)	57 (0.14)	41 (0.14)	48 (0.14)	51 (0.13)	197 (0.14)
Identified poppers consumption	25 (10.46)	2 (4.76)	9 (15.79)	3 (7.32)	5 (7.32)	6 (11.76)	23 (11.67)
≥5% and <25% Identified poppers consumption	220 (92.05) 12/220	40 (95.24)	52 (91.23)	37 (90.24)	46 (95.83)	45 (88.24)	180 (91.37)
≥25% Identified poppers consumption	19 (7.95) 13/19	2 (4.76)	5 (8.77)	4 (9.76)	2 (4.17)	6 (11.76)	17 (8.63)

MetHbia: methemoglobinemia.

**Table 2:** Description of the characteristics of cases of poppers consumers according to MetHbia rates (n = 47).

	Total Poppers Consumers (n = 47)	Consumers with MetHbia <25% (n = 20)	Consumers with MetHbia ≥25% (n = 27)
Male Sex, n (%)	42 (89.4)	18 (90)	24 (88.9)
Age (years), mean (sd) Min–Max	35 (10.3) 19–54	34 (9.9) 19–54	36 (10.5) 19–52
Median of maximum MetHbia level per patient [IQR] Min–Max	25.1% [20.8–37.7%] 5.4–87.8%	17.6% [12.625–23.15%] 5.4–24.9%	31.7% [27–54.4%] ^b^ 25–87.8%
Mode of administration			
Inhaled only ^a^, n (%) Median of maximum MetHbia level per patient [IQR] Min–Max	37 (78.7) 25.0% [20.8–28.6%] 5.4–87.8%	18 (90.0) 19.7% [13.375–23.45%] 5.4–24.9%	19 (70.4) 28.6% [26.5–48.85%] 25–87.8%
Oral only, n (%) Median of maximum MetHbia level per patient [IQR] Min–Max	9 (19.1) 30.6% [20–57.35%] 11.5–76.9%	2 (10.0) 12.3% [11.875–12.625%] 11.5–13%	7 (25.9) 38.8% [30.6–75.9%] ^b^ 27–76.9%
Combined oral and inhaled, n (%) Median of maximum MetHbia level per patient	1 (2.1) 31.2%	0	1 (3.7) 31.2%
Associated substances consumption, n (%) At least one substance Alcohol Amphetamines Sildenafil Benzodiazepines Cocaine Cannabis NPS	16 (30.0) 13 (27.7) 4 (8.5) 2 (4.3) 1 (2.1) 1 (2.1) 1 (2.1) 1 (2.1)	5 (25.0) 4 (20.0) 2 (10.0) 0 0 0 1 (5.0) 1 (5.0)	11 (40.7) 9 (33.3) 2 (7.4) 2 (7.4) 1 (3.7) 1 (3.7) 0 0
Symptoms, n (%) Cyanosis and/or discoloration Desaturation Respiratory dysfunction Consciousness disorder Malaise Coma Cardiac dysfunction Psychiatric symptoms Dizziness	32 (68.1) 21 (44.7) 13 (27.7) 12 (25.5) 8 (17.0) 5 (10.6) 6 (12.8) 2 (4.25) ^c^ 2 (4.25)	9 (45.0) 8 (40.0) 5 (25.0) 1 (5.0) 1 (5.0) 2 (10.0) 3 (15.0) 2 (10.0) 0	23 (85.2) 13 (48.1) 8 (29.6) 11 (40.7) 7 (25.9) 3 (11.1) 3 (11.1) 0 2 (7.4)
Complications n (%) Rhabdomyolysis Hemolytic anemia Serotoninergic syndrome Pulmonary embolism Syncope Cardiorespiratory arrest Toxic encephalopathy	8 (17.0) 2 (4.3) 2 (4.3) 2 (4.3) 1 (2.1) 1 (2.1) 1 (2.1) 1 (2.1)	6 (30.0) 2 (10.0) 2 (10.0) 2 (10.0) 1 (5.0) 0 0 1 (5.0)	2 (7.4) 0 0 0 0 1 (3.7) 1 (3.7) 0
Support, n (%) Methylene blue Oxygen therapy Specialized department Simple monitoring or rehydration Unspecified	32 (68.1) 27 (57.4) 10 (21.3) 5 (10.6) 1 (2.1)	11 (55.0) 10 (50.0) 7 (35.0) 3 (15.0) 0	21 (77.8) 17 (62.9) 3 (11.1) 2 (7.4) 1 (3.7)
Evolution, n (%) Favorable Deceased Unspecified	43 (91.5) 1 (2.1) 3 (6.4)	19 (95.0) 0 1 (5.0)	24 (88.9) 1 (3.7) 2 (7.4)

IQR: interquartile range. Sd: standard deviation. ^a^: When the mode of administration was missing, it was considered as inhaled as it is the most frequent mode of administration for poppers. ^b^: 2 missing values for MetHbia level above 25% (concentration above the saturation threshold of the device used). ^c^: Both cases including consumption of another psychoactive drug. MetHbia: methemoglobinemia. NPS: new psychoactive substances.

## Data Availability

Data is contained within the article.
